# Altered Resting-State Brain Activities in Drug-Naïve Major Depressive Disorder Assessed by fMRI: Associations With Somatic Symptoms Defined by *Yin*-*Yang* Theory of the Traditional Chinese Medicine

**DOI:** 10.3389/fpsyt.2018.00195

**Published:** 2018-05-15

**Authors:** Zhexue Xu, Shu Zhang, Liyuan Huang, Xiaolei Zhu, Qing Zhao, Yawei Zeng, Dongfeng Zhou, Di Wang, Hironori Kuga, Atsushi Kamiya, Miao Qu

**Affiliations:** ^1^Department of Neurology, Xuan Wu Hospital, Capital Medical University, Beijing, China; ^2^Third Affiliated Hospital, Beijing University of Chinese Medicine, Beijing, China; ^3^Department of Neurology, Fengtai Integrated Chinese and Western Medicine Hospital of Beijing, Beijing, China; ^4^Department of Psychiatry and Behavioral Sciences, Johns Hopkins University School of Medicine, Baltimore, MD, United States; ^5^School of Applied Psychology and Menzies Health Institute Queensland, Griffith University, Brisbane, QLD, Australia; ^6^Department of Radiology, People's Liberation Army No.306 Hospital, Beijing, China; ^7^Department of Clinical Psychology, Peking University Sixth Hospital, Beijing, China; ^8^Department of Clinical Psychology, Beijing Anding Hospital, Beijing, China; ^9^Johns Hopkins Bloomberg School of Public Health, Baltimore, MD, United States

**Keywords:** major depressive disorder, somatic symptoms, resting-state fMRI, *Yin*-*Yang* theory, traditional Chinese medicine

## Abstract

Identification of biological markers for defining subtypes of major depressive disorder (MDD) is critical for better understanding MDD pathophysiology and finding effective treatment intervention. The “*Yin* and *Yang*” theory is a fundamental concept of traditional Chinese Medicine (TCM). The theory differentiates MDD patients into two subtypes, *Yin* and *Yang*, based on their somatic symptoms, which had empirically been used for the delivery of effective treatment in East Asia. Nonetheless, neural processes underlying *Yin* and *Yang* types in MDD are poorly understood. In this study, we aim to provide physiological evidence using functional magnetic resonance imaging (fMRI) to identify altered resting-state brain activity associated with *Yin* and *Yang* types in drug-naïve MDD patients. The *Yin* type and *Yang* type MDD patients showed increased amplitude of low-frequency fluctuation (ALFF) in different cortical brain areas in the parietal, temporal, and frontal lobe, compared to matched healthy controls. Differential ALFF is also observed in several cortical areas in frontal lobe and insula between *Yin* and *Yang* type group. Of note, although ALFF is increased in the inferior parietal lobe in both *Yin* and *Yang* type group, inferior parietal lobe-centered functional connectivity (FC) is increased in *Yang* type, but is decreased in *Ying* type, compared with matched healthy controls. These results suggest that differential resting-state brain activity and functional connectivity in *Yin* and *Yang* types may contribute to biological measures for better stratification of heterogeneous MDD patients.

## Introduction

Major depressive disorder (MDD) is a prevalent psychiatric condition associated with increased mortality and healthcare resource utilization, imposing a serious economic burden on our society (1, 2). MDD is defined based on clinical manifestation utilized in current gold standard diagnostic classification systems such as Statistical Manual of Mental Disorders (DSM) and International Statistical Classification of Diseases and Related Health Problems (ICD) (3, 4). Although these categorical frameworks contribute to the advancement of biological understanding and patient care, the pathophysiology of MDD remains elusive. Moreover, many MDD patients exhibit treatment-resistance to current antidepressants, potentially due to its heterogeneous nature with multiple etiologies (5).

Considering these issues, many studies attempt to identify biological and physiological markers to differentiate subtypes of MDD, which may be more responsive to specific treatment approaches (6–10). For instance, Raison and colleagues reported that infliximab, a tumor necrosis factor (TNF) antagonist, ameliorates depressive symptoms in a subpopulation of MDD patients who have high level of C-reactive protein (CRP) in plasma, but not in those with low levels of CRP (6). Drysdale et al. have recently demonstrated that different patterns of altered neural connectivity involved in limbic and frontostriatal regions can classify MDD patients into four physiological subtypes, so-called biotypes (8). Interestingly, these biotypes predict effectiveness of treatment with transcranial magnetic stimulation.

Traditional Chinese Medicine (TCM), developed a few thousand years ago, approaches diagnosis and treatment differently from Western medicine (11, 12). For example, patients are classified into two subgroups based on their somatic symptoms according to the “*Yin* and *Yang*” theory, the fundamental concept of the TCM. The “*Yin* and *Yang*” theory has historically been documented in the TCM book, entitled *Huangdi Neijing*, which was written over two thousand years ago in China (13). *Yin* patients are characterized by an intolerance to cold with a preference to warm environments and hot beverages and food, whereas *Yang* patients are characterized by an intolerance to heat with a preference to cool environments, cold beverages, and food. Although current diagnostic criteria, such as DSM-5 and ICD-10, do not consider somatic symptoms for diagnosis of MDD, the contrastive *Yin* and *Yang* phenotypes might be useful for identifying biotypes of MDD. Nonetheless, physiological neural processes underlying *Yin* and *Yang* types in MDD remain poorly explored.

The aim of this exploratory study is to provide physiological evidence, using functional magnetic resonance imaging (fMRI), to identify altered resting-state brain activity associated with *Yin* and *Yang* types in drug-naïve MDD patients. Previous studies using functional connectivity (FC) method of resting-sate fMRI demonstrated altered inter- and intral-regional brain connectivity, including local functional connectivity in the medial prefrontal cortex and frontoparietal hypoconnectivity in MDD brains (14, 15). As proposed in Drysdale's work (8), differential brain function at resting-state may be a useful physiological marker to identify specific subpopulation of MDD patients. Thus, we hypothesize that resting-state brain activity and FC in MDD patients with *Yin* type are altered when compared to those with *Yang* type. To test this hypothesis, we examined resting-state functional activities across the entire brain in MDD patients in both *Yin* and *Yang* groups as well as matched healthy controls.

## Materials and methods

### Participants

Patients with MDD were recruited from the outpatient clinic of The Third Affiliated Hospital of Beijing University of Chinese Medicine, Peking University Sixth Hospital, and Beijing Anding Hospital. Healthy controls were recruited from community-based advertising through flyers posted at hospital and university campuses. All participant-involved study activities were approved by the Medical Research Ethics Committee of The Third Affiliated Hospital of Beijing University of Chinese Medicine. All participants were recruited from October 2015 to December 2016. Exclusion criteria for MDD patient group and healthy controls were: (1) left-handedness; (2) age <18 years and >45 years; (3) any neurological illness; (4) history of any other major psychiatric disease; (5) any diagnosed physical disease; (6) any brain white matter changes detected by T2-weigheted magnetic resonance images; (7) metallic foreign bodies, pacemakers, metallic dentures, or amalgam fillings; (8) females in a post-menopausal and menopausal condition. MDD patients also met the following additional inclusion criteria: (1) diagnosis of moderate depression; (2) 17-item Hamilton Depression Rating scale (HAM-D) scores ≥17 and < 24 and Young Mania Rating scale (YMRS) scores < 5; (3) no diagnosis for any other mental disorders according to DSM-IV criteria; (4) medication-free for at least 2 weeks. The additional inclusion criteria for healthy controls was HAM-D score < 7. To control study variance, MDD subjects were matched individually to one healthy control subject for gender, age, education years, and IQ level.

### Clinical measures

All participants underwent structured clinical interview and were diagnosed by two well-trained senior psychiatrists according to the criteria defined by the DSM-IV. Clinical symptoms were measured on the same day as the fMRI measurement. HAM-D (16) and YMRS (17) were used to assess psychiatric symptoms. Three well-experienced physician scientists classified patients in *Yin* and *Yang* types based on somatic symptoms that occurred no <2 weeks before recruitment and the results of *Yin* and *Yang* questionnaires (Supplementary Table 1). The *Yang* group feels hot or has hot flashes, sweats during daily activities, and prefers cool environments and cold beverages and food. The *Yin* group feels cold or has cold flashes, does not sweat even during strenuous exercise, and prefers warm environments and hot beverages and food (18). We also confirmed that matched healthy control subjects had no somatic symptoms. The *Yin-Yang* Questionnaire was established by our research group, consisting of three psychiatrists and three physician scientists of traditional Chinese medicine. The reliability of this questionnaire was initially tested in 30 Yang and 30 Yin depression patients (Cronbach's Alpha was 0.861, and 0.823, respectively). In this questionnaire, we weight patients' subjective symptoms as primary scores, objective physical signs as secondary scores, and use a set of 5 levels to evaluate the degree of symptoms. In order to exclude the comorbidity of *Yin* and *Yang* types, we also set cut-off points of *Yin* and *Yang*.

### MRI data acquisition

T1-weighted and resting-state fMRI data were acquired using a 3T Siemens Trio scanner (Magnetom Allegra, Siemens, Erlangen, Germany) in the Beijing PLA 306 Hospital. A high-resolution image covering the whole brain was acquired with the following scan parameters: Repetition Time (TR)/Echo Time (TE) = 2,300/3.0 ms, flip angle = 90°, Field of view (FOV) = 240 × 256 mm^2^, voxel size = 1.0 × 1.0 × 1.0 mm^3^, 176 sagittal slices, duration = 6 min 06 s. Resting-state functional imaging scans contain 180 functional volumes, using a T2-weigted Echo Planar Imaging sequence with the following parameters: TR/TE = 2,000/30 ms, flip angle = 90°, acquisition matrix = 64 × 64, in-plane resolution 3.0 × 3.0 mm^2^, FOV = 210 × 210 mm^2^, axial slices = 32, thickness/gap = 4/0 mm, the total sequence time is 6 min 06 s. During the scanning process, patients were monitored to ensure that they remained awake. Immediately following the scans, a simple inquiry was conducted to exclude any sleeping periods as well as any uncomfortableness felt by the participant during the examination.

### Resting-state fMRI data processing

Image preprocessing and statistical analyses were performed using the Data Processing Assistant for Resting-state fMRI (DPARSF, http://www.restfmri.net/forum/DPARSF) toolkits ^20^ and SPM8 software (SPM8, http://www.fil.ion.ucl.ac.uk/spm/). All images were drafted by BrainNet Viewer toolkit (19).

#### Pre-processing

Data pre-processing was performed by standard procedures in DPARSF toolkits. Images were pre-processed according to the following steps: the original DICOM data were converted to the NifTI format; the first 10 images were discarded to allow for instrumental stabilization of the initial signal; the images were slice-timed and realigned to correct for head motions; Patient's and healthy control's data were excluded if movement in the translational or rotational planes exceeded 3 mm or 3° and 1 mm or 1°, respectively; the images were normalized based on the Montreal Neurological Institute (MNI) Space with Smoothing Method (Full Width at Half Maximum, FWHM 6 mm); resting-state fMRI data were processed with linear detrending and 0.01–0.08 Hz band-pass filtering.

#### ALFF and FC analysis

Using Resting-State fMRI Data Analysis Toolkit (REST) 1.6 software (http://restfmri.net/forum/REST) (20), linear tendency of the data after pre-processing (space smoothing was completed) was removed by linear regression. After pre-processing, the time series for each voxel was first filtered (band-pass, 0.01–0.08 Hz) to remove the effects of very low-frequency drift and high-frequency noise, and then converted to the frequency domain using a Fast Fourier Transform (FFT). The square root of the power spectrum was computed. This averaged square root was termed ALFF at the given voxel (21). In the following FC analysis, brain regions that showed significant ALFF changes were set as seeds to examine whole-brain functional connectivity across all brain regions.

### Statistical analysis

Participants' demographic information, including age, gender, education level and IQ, as well as was compared between *Yin* and *Yang* MDD groups and their matched health control groups using two samples *t*-tests. Chi-square tests were applied to detect gender related differences. The statistical significance level was set at *p* < 0.05. All statistical tests were performed using SPSS 18.0 (SPSS Inc., Chicago, IL, USA). Pearson correlation coefficients were calculated to assess the relationship between ALFF of regions of interest and HAM-D total scores or its sub-factors. We did not apply corrections for multiple tests in this Pearson correlation coefficients because the analyses were considered exploratory in nature. The fMRI data were analyzed by technologists who were blind to the diagnosis of the participants, in the National Key Laboratory of Cognitive Neuroscience and Learning of Beijing Normal University. One-sample *t*-test was computed for significant brain activation in whole brain in every subject using REST 1.6 software (Threshold *p* < 0.05). Voxel-wise group comparisons were detected with two-sample *t*-test (AlphaSim correction *p* < 0.01; continuous voxels >71). REST 1.6 software Viewer was applied to identify the precise anatomical position in the brain with statistical significance on the corresponding MNI coordinate. Voxel-wise FC analyses revealed the Pearson correlation coefficients between the seeds and the rest of the whole brain areas. FC values were transformed into *z*-values by using Fisher *r*-to-*z* transformation. Two-sample *t*-tests were used to disclose the group differences in the functional connectivity (AlphaSim correction *p* < 0.01; continuous voxels >71).

## Results

### Demographic and clinical characteristics of the study groups

MDD patients (*n* = 12 *Yin* and *n* = 12 *Yang* group) and matched healthy controls (*n* = 12 for *Yin* and *Yang* MDD group, respectively) participated in this study. The demographic information for these four groups was shown in Table 1. There were no statistical differences in age, gender, years of education, and IQ between *Yin* and *Yang* types MDD patient and healthy control groups. The *Yin* and *Yang* types MDD patients scored higher on the *Yin* and *Yang* questionnaires compared to their matched healthy controls (*Yin*: 41.08 ± 3.58 vs. 2.50 ± 1.38, *p* < 0.01; *Yang*: 39.58 ± 3.45 vs. 1.92 ± 1.73, *p* < 0.01). As shown in Table 2, the *Yin* and *Yang* type MDD patient groups had higher HAM-D scores compared to those of their healthy controls (*Yin*: 23.67 ± 2.57 vs. 0.92 ± 0.90; *Yang*: 20.58 ± 2.02 vs. 0.83 ± 0.84, *p* < 0.01). The HAM-D score of *Yin* type MDD patients was higher than that of *Yang* type MDD patients (23.67 ± 2.57 vs. 20.58 ± 2.02, *p* < 0.01). In the HAM-D factors, cognitive disturbance was severe in *Yin* type MDD patients compared with those in *Yang* type MDD patients (6.83 ± 1.85 vs. 5.92 ± 0.99, *p* < 0.05), whereas there was no difference in psychomotor retardation and dyssomnia between groups.

**Table 1:** Demographic and clinical information for the participants.

	***Yin* (*n* = 12)**	***Yang* (*n* = 12)**	***Yin*-HC (*n* = 12)**	***Yang*-HC (*n* = 12)**	***t*****/*****p*****/*****χ^**2**^***		
					***Yin* vs. *Yang***	***Yin* vs. *Yin-HC***	***Yang* vs. *Yang-HC***
Age (mean, *SD*)	29.83 ± 6.97	32.67 ± 6.30	29.91 ± 6.97	32.17 ± 6.26	*t* = 1.03	*t* = −0.03	*t* = 0.19
					*p* = 0.32	*p* = 0.98	*p* = 0.85
Gender (male/female)	7/5	5/7	7/5	5/7	χ^2^ = 0.67	χ^2^ = 0.00	χ^2^ = 0.00
					*p* = 0.68	*p* = 1.00	*p* = 1.00
Education (mean, *SD*)	16.50 ± 2.81	17.17 ± 4.06	16.50 ± 2.81	17.17 ± 4.06	*t* = 0.47	*t* = 0.00	*t* = 0.00
					*p* = 0.65	*p* = 1.00	*p* = 1.00
IQ (mean, *SD*)	99.50 ± 6.82	99.41 ± 7.32	101.75 ± 8.25	100.42 ± 8.37	*t* = −0.29	*t* = −7.28	*t* = −3.12
					*p* = 0.98	*p =* 0.47	*p =* 0.76
Somatic Symptoms (mean, *SD*)	41.08 ± 3.58	39.58 ± 3.45	2.50 ± 1.38	1.92 ± 1.73		*t* = 34.84	*t* = 33.81
						*p* < 0.01	*p* < 0.01

*Yin, Yin type MDD patients; Yang, Yang type MDD patients; HC, matched healthy controls; IQ, Intelligence Quotient; Somatic Symptoms, the Yang/Yin scores; SD, standard deviation*.

**Table 2:** The total HAMD and sub-factors scores information for the participant.

	***Yin* (*n* = 12)**	***Yang* (*n* = 12)**	***Yin*-HC (*n* = 12)**	***Yang*-HC (*n* = 12)**	***t*****/*****p*****/**χ^**2**^		
					***Yin* vs. *Yang***	***Yin* vs. *Yin-HC***	***Yang* vs. *Yang-HC***
HAMD score (mean, *SD*)	23.67 ± 2.57	20.58 ± 2.02	0.92 ± 0.90	0.83 ± 0.84	*t* = −3.27	*t* = 28.94	*t* = 31.29
					*p* < 0.01	*p* < 0.01	*p* < 0.01
Anxiety/Somatization (mean, *SD*)	6.17 ± 1.95	5.75 ± 1.22	0.58 ± 0.67	0.50 ± 0.67	*t* = −0.63	*t* = 9.40	*t* = 13.09
					*p* = 0.54	*p* < 0.01	*p* < 0.01
Weight (mean, *SD*)	0.50 ± 0.52	1.00 ± 0.74	0.00 ± 0.00	0.00 ± 0.00	*t* = 1.92	*t* = 3.32	*t* = 4.69
					*p* = 0.07	*p* < 0.01	*p* < 0.01
Cognitive disturbance (mean, *SD*)	5.00 ± 1.54	3.67 ± 0.89	0.00 ± 0.00	0.08 ± 0.29	*t* = −2.60	*t* = 11.27	*t* = 13.30
					*p* = 0.02	*p* < 0.01	*p* < 0.01
Retardation (mean, *SD*)	6.83 ± 1.85	5.92 ± 0.99	0.00 ± 0.00	0.00 ± 0.00	*t* = −1.51	*t* = 12.80	*t* = 20.57
					*p* = 0.15	*p* < 0.01	*p* < 0.01
Dyssomnia (mean, *SD*)	3.50 ± 1.51	2.58 ± 1.31	0.33 ± 0.49	0.17 ± 0.39	*t* = −1.59	*t* = 6.92	*t* = 6.12
					*p* = 0.13	*p* < 0.01	*p* < 0.01

*Yin, Yin type MDD patients; Yang, Yang type MDD patients; HC, matched healthy controls; HAMD, Hamilton Depression Rating scale; SD, standard deviation. The HAMD scale was classified by dividing items into 5 sub-scales and determining the average score of each item, as a score of sub-factors, as follows: Anxiety/Somatization: average of scores for anxiety psychic; anxiety somatic; somatic symptoms gastro-intestinal; general somatic symptoms; insight. Weight: loss of weight. Cognitive disturbance: average of scores for feelings of guilt; suicide; agitation. Retardation: average of scores for depressed mood; work and activities; retardation; genital symptoms. Dyssomnia: average of scores for insomnia early in the night; Insomnia middle of the night; Insomnia early hours of the morning*.

### ALFF analysis

**Figure 1:**
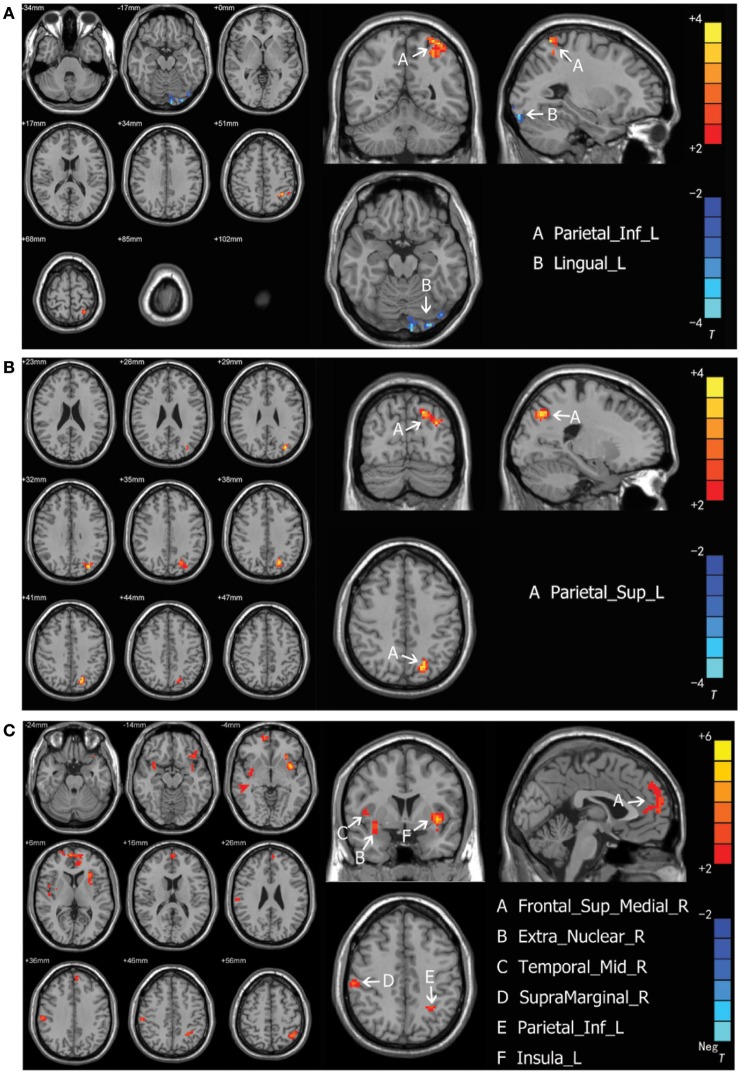
**(A)** Brain activation regions of rs-fMRI using Amplitude of Low-Frequency Fluctuation (ALFF) method in depression patients compared with the entire normal control cohort. A Parietal_Inf_L; left inferior parietal extending to supramarginal and angular gyri, B Lingual_L; left lingual gyrus. **(B)** Brain activation regions of rs-fMRI using Amplitude of Low-Frequency Fluctuation (ALFF) method in *Yin* type compared with matched healthy controls. A Parietal_Sup_L; left superior parietal gyrus. **(C)** Brain activation regions of rs-fMRI using Amplitude of Low-Frequency Fluctuation (ALFF) method in *Yang* type compared with matched healthy controls. A Frontal_Sup_Medial_R; right superior frontal gyrus, extending to medial, B Extra_Nuclear_R; right extra-nuclear, C Temporal_Mid_R; right middle temporal gyrus; D SupraMarginal_R; right supramarginal gyrus, E Parietal_Inf_L; left inferior parietal, extending to supramarginal and angular gyri, F Insula_L; left insula.

Intergroup differences of results from ALFF analysis were shown in Table 3. Compared to the healthy control group, MDD patients group showed increased ALFF in left inferior parietal, extending to supramarginal and angular gyri (−45, −51, 57. BA40/7) and decreased ALFF in left lingual gyrus (−27, −93, −18. BA18) (Figure 1A). Increased ALFF in *Yin* type MDD patients compared to their healthy controls was only observed in left superior parietal gyrus (−21, −72, 42. BA7/19) (Figure 1B). An increase in ALFF were found in *Yang* type MDD patient group compared to their healthy controls in the right superior frontal gyrus, extending to medial (6, 60, 3. BA10/9/32), the right middle temporal gyrus (54, −27, −9. BA21), the right extra-nuclear (33, 3, −12. BA13), the right supramarginal gyrus (60, −24, 36. BA2/3/1), the left inferior parietal, extending to supramarginal and angular gyri (−39, −57, 57. BA40/7), and the left insula (−36, 6, −6. BA13/47) (Figure 1C).

**Table 3:** The comparison of regional brain activity in *Yin* and *Yang* type MDD patients and matched controls (AlphaSim-corrected, *p* < 0.01).

**Brain Areas**			**BA**	**Voxels**	**MNI**			***T*-scores**
**Hemisphere**	**Region**	**Label**			**x**	**y**	**z**	
**(*****Yin*** + ***Yang*** > **All HC)**								
Left	Parietal	Inferior parietal, extending to supramarginal and angular	40/7	107	−45	−51	57	3.55
**(*****Yin*** + ***Yang*** <**All HC)**								
Left	Occipital	Lingual gyrus	18	80	−27	−93	−18	−3.29
**(*****Yin*** > ***Yin*****-HC)**								
Left	Parietal	Superior parietal gyrus	7/19	91	−21	−72	42	4.41
**(*****Yang*** > ***Yang*****-HC)**								
Right	Frontal	Superior frontal gyrus, extending to medial	10/9/32	199	6	60	3	5.08
Right	Temporal	Middle temporal gyrus	21	74	54	−27	−9	4.04
Right	Temporal	Extra-nuclear	13	101	33	3	−12	3.40
Right	Parietal	Supramarginal gyrus	2/3/1	76	60	−24	36	3.72
Left	Parietal	Inferior parietal, extending to supramarginal and angular	40/7	83	−39	−57	57	4.11
Left	Insula	Insula	13/47	205	−36	6	−6	5.73
**(*****Yang*** > ***Yin*****)**								
Left	Frontal	Superior frontal gyrus, extending to medial	10/32/11/9	276	−6	57	0	4.94
Left	Frontal	Median cingulate and paracingulate gyri	32/24	85	0	15	39	4.11
Left	Insula	Insula	13	128	−33	21	6	4.64

*Yin, Yin type MDD patients; Yang, Yang type MDD patients; HC, matched healthy controls; MNI: Montreal Neurological Institute; x, y, and z: coordinates of primary peak locations in the MNI space*.

**Figure 2:**
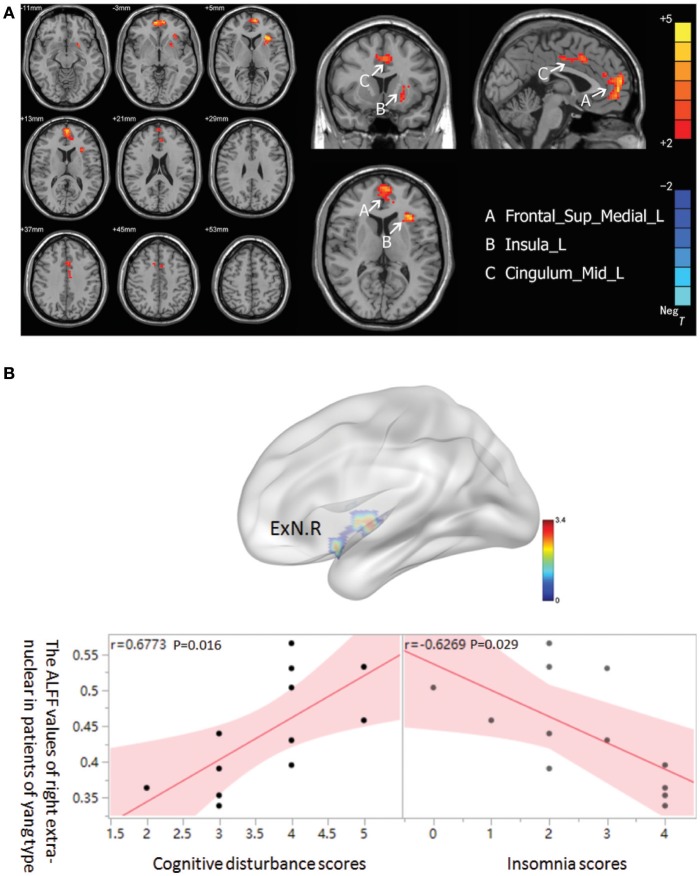
**(A)** Brain activation regions of rs-fMRI using Amplitude of Low-Frequency Fluctuation (ALFF) method in *Yang* type compared with *Yin* type. A Frontal_Sup_Medial_R; left superior frontal gyrus, extending to medial, B Insula_L; left insula, C Cingulum_Mid_L; left median cingulated and paracingulate gyri. **(B)** The correlation between ALFF of right extra-nuclear and two sub-factors of HAMD in *Yang* type MDD patients. The graph indicates that the ALFF of right extra-nuclear (ExN.R) had positive correlation with cognitive disturbance and negative correlation with insomnia.

In the MDD patients group, increased ALFF was observed in *Yang* type group compared to *Yin* type group in the left superior frontal gyrus, extending to medial (−6, 57, 0. BA10/32/11/9), the left median cingulate and paracingulate gyri (0, 15, 39. BA32/24), and the left insula (−33, 21, 6. BA13) (Figure 2A). There was no significant correlation between ALFF values and HAM-D scores in either *Yin* and *Yang* type of MDD patients. Nonetheless, we found that the ALFF value of the right extra-nuclear in the *Yang* type group was positively correlated with cognitive disturbance (*r* = 0.6773, 0.95 CI: 0.169–0.901; *p* = 0.016), and was negatively correlated with insomnia (*r* = 0.6269, 0.95 CI: −0.883 to −0.083; *p* = 0.029) in sub-factor analysis (Figure 2B). We also analyzed the correlation between the Yin and Yang scores and ALFF of brain regions. Although we observed a negative correlation between Yang scores and left insula (*r* = −0.577 *p* = 0.050), there is no significant correlation after FDR correction (*p* = 0.3). We also found no significant correlation of Yin score and ALFF.

### FC analysis

**Figure 3:**
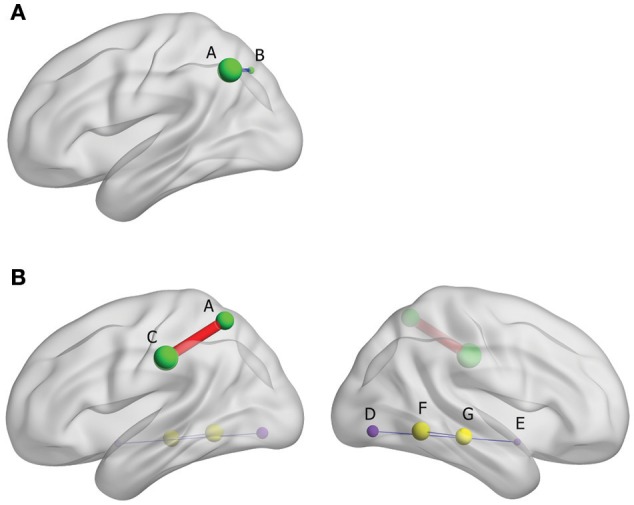
**(A)** Brain functional connectivity of rs-fMRI in *Yin* type compared with healthy controls; size of balls represent the t-scores of every brain regions; blue thin edge represents the negative FC between the different regions; A: left inferior parietal, extending to supramarginal and angular gyri, B: left superior parietal gyrus. **(B)** Brain functional connectivity of rs-fMRI in *Yang* type compared with healthy controls; size of balls represents the t-scores of every brain regions; red bold edge represents the positive FC between the different regions, conversely, blue thin edges represent the negative FC; same colored ball represents connecting brain region; A: left inferior parietal, extending to supramarginal and angular gyri, C: left supramarginal gyrus, D: right extra-nuclear, E: right lingual gyurs, F: right middle temporal gyrus, G: Fusiform gyrus.

The results from FC analysis, indicating intergroup differences, are shown in Table 4. The significantly changed ALFF in *Yin* and *Yang* types compared with matched healthy controls were taken as seeds. In *Yin* type patients, negative FC was observed between left superior parietal gyrus and left inferior parietal, extending to supramarginal and angular gyri (−48, −60, 42. BA40; Figure 3A). In *Yang* type patients, the left inferior parietal, extending to supramarginal and angular gyri showed positive FC with left supramarginal gyrus (−57, −24, 36. BA2/40), whereas right extra-nuclear and right middle temporal gyrus showed negative FC with right lingual gyrus (24, −78, −6. BA17) and right fusiform gyrus (30, −51, −6. BA19), respectively (Figure 3B).

**Table 4:** The comparison of functional connectivity in *Yin* and *Yang* type MDD patients with matched controls (AlphaSim-corrected, *p* < 0.01).

**Regions of interest**	**Brain areas**			**BA**	**Voxels**	**MNI**			***T*-scores**	***z*-scores**
	**Hemisphere**	**Region**	**Label**			**x**	**y**	**z**		
(***Yin*****-HC** > ***Yin*****)**										
Left superior parietal gyrus	Left	Parietal	Inferior parietal, extending to supramarginal and angular	40	84	−48	−60	42	−3.96	0.22
**(*****Yang*** > ***Yang*****-HC)**										
Left Inferior parietal, extending to supramarginal and angular	Left	Parietal	Supramarginal gyrus	2/40	145	−57	−24	36	4.53	0.57
**(*****Yang*****-HC** > ***Yang*****)**										
Right middle temporal gyrus	Right	Temporal/Occipital	Fusiform gyrus	17	176	30	−51	−6	−4.11	0.17
Right extra-nuclear	Right	Occipital	Lingual gyrus	19	104	24	−78	−6	−3.75	0.22

*Yin, Yin type MDD patients; Yang, Yang type MDD patients; HC, matched healthy controls; MNI, Montreal Neurological Institute; x, y, and z: coordinates of primary peak locations in the MNI space*.

## Discussion

The recent meta-analysis of resting-state fMRI studies demonstrated altered neural connectivity in several brain regions in MDD patients (15). These include hypoconnectivity of frontoparietal systems and dorsal attention network, which are critical brain regions for regulating mood, emotion, and attention (22–25). Another meta-analysis of resting-state fMRI studies showed altered local functional connectivity in the medial prefrontal cortex in drug-naïve MDD patients (14). Consistent with previous studies (26–29), we found increased resting-state brain activity in left inferior parietal, extending to supramarginal gyrus, and decreased activation in left lingual gyrus in MDD patients compared to the healthy control group. We also observed increased activation in angular gyrus, whereas the previous study reported decreased activity in this area in patients with MDD (30). Note that considering heterogeneous etiology and symptomatology of MDD, we focused on drug-naïve MDD patients with moderate symptoms in the present study. Thus, the discrepancy between these results may be explained by different criteria of patient recruitment.

To the best of our knowledge, this is the first study demonstrating biological differences in brain function associated with *Yin* and *Yang* types characterized by somatic symptoms. The “*Yin* and *Yang”* theory that had originally been developed in the ancient China, is utilized for patient treatment in TCM (31–34). Western modern science has recently begun to explore biological significance of “*Yin* and *Yang”* types. For instance, the *Yin* and *Yang* types are associated with genetic mutations in epidermal growth factor receptor (*EGFR*) in patients with non-small cell lung cancer (18). We observed increased resting-state brain activity in *Yang* type patients in the left superior frontal gyrus, extending to medial, the left median cingulate and paracingulate gyri, and the left insula compared to *Yin* type group. The resting-state activity of these brain regions was also increased in *Yang* type patients compared to their healthy controls, but not in *Yin* type patients. In addition to mood and emotion, these regions are critical for cognitive processing (35–37), thus their differential activity may contribute to better cognitive performance in *Yang* Type, compared with *Yin* type. We also found that the ALFF value of the right extra-nuclear in the *Yang* type group was positively correlated with cognitive disturbance, and was negatively correlated with insomnia. Interestingly, the extra-nuclear is a part of the ventral affective processing system, including subcortical areas such as the hippocampus, amygdala, and thalamus. Importantly, previous studies reported the importance of these subcortical areas in the control of cognitive processing and sleep regulation (38–43). While ALFF in the inferior parietal lobe is increased in both *Yin* and *Yang* type group, we found different FC patterns between *Yin* and *Yang* type group in this brain area. Inferior parietal lobe-centered FC is increased in *Yang* type, but is decreased in *Ying* type, compared with matched healthy controls, suggesting that differential FC may be useful in physiologically differentiating *Yin* and *Yang* type MDD patients.

Somatic symptoms may, in part, be mediated by imbalance between sympathetic nervous system (SNS) and parasympathetic nervous system (PNS) (44, 45). As mentioned above, specific brain areas differentially activated in *Yin* and *Yang* types are involved in emotional processing which may affect sympathetic and parasympathetic neuronal activities (46, 47). Notably, several neuroimaging studies suggest that insular cortex plays a role for thermoregulatory mechanisms to exogenous thermal stimulation (48–50). To delineate differential brain function in *Yin* and *Yang* types, it would be of great interest to further examine neural mechanisms underlying the impact of imbalance of SNS and PNS on somatic symptoms.

## Limitations

There are several limitations of this study. First, as we recruited only moderate MDD patients, selection bias should be considered. Mild and severe MDD patients should be examined by the same approach to determine if the altered neural activity that we observed is a state-dependent endophenotype. Second, given that the number of participants was small, the statistical power for assessing brain activity is limited. Future research, with recruitment of a larger sample size, is needed to determine if resting-state brain activity can differentiate *Yin* and *Yang* types in MDD. It is also important to examine whether clinical manifestation using *Yin* and *Yang*” theory may differentiate brain function in other depressive conditions. Third, although three well-experienced physician scientists classified patients in *Yin* and *Yang* types based on somatic symptoms, establishing a standardized clinical assessment tool of *Yin* and *Yan* types is necessary for future studies. Nevertheless, this line of research may contribute to the understanding of the neural basis of *Yin* and *Yang* types, which may provide a step toward evidence-based application of “*Yin* and *Yang”* theory in modern Western medicine.

## Conclusion

The “*Yin* and *Yang”* theory has long time been utilized for patient treatment in the field of TCM. However, lack of evidence of the underlying biological mechanisms hampers its use in modern Western medicine. Our study revealed potential differential resting-state brain activation in *Yin* and *Yang* types in drug-naïve MDD patients. Understanding the neural mechanism underlying somatic symptoms may contribute to biological measure for better stratification of heterogeneous MDD patients, which might improve treatment approaches.

## Ethics statement

This study was carried out in accordance with the recommendations of the Ethical Committee at the Third Affiliated Hospital of Beijing University of Chinese Medicine. The protocol (KTPJ-BZYSY-2015-13) was approved by the Ethical Committee at the Third Affiliated Hospital of Beijing University of Chinese Medicine. All subjects gave written informed consent in accordance with the Declaration of Helsinki.

## Author contributions

ZX and SZ: participated in the design of the study, conducted the analyses, and wrote the manuscript; SZ: collected the clinical information and performed the TCM syndrome assessment; LH and XZ: helped with the design and coordination of the study and wrote the manuscript; YZ: participated in fMRI data collection; DZ and DW: helped collected depression patients; QZ, HK, and AK: contributed to interpretation of the data and drafting the manuscript; MQ: conceived and coordinated the design of the study, and wrote the manuscript. All authors read and approved the final manuscript.

### Conflict of interest statement

The authors declare that the research was conducted in the absence of any commercial or financial relationships that could be construed as a potential conflict of interest.
