# Supplementary material for: Altered Resting-State Brain Activities in Drug-Naïve Major Depressive Disorder Assessed by fMRI: Associations With Somatic Symptoms Defined by Yin-Yang Theory of the Traditional Chinese Medicine
Source: Front Psychiatry. 2018 May 15;9:195. doi: 10.3389/fpsyt.2018.00195 (PMC5962703; doi:10.3389/fpsyt.2018.00195)
Supplement: Supplementary file 1 [file Table_1.doc]

**Supplementary Table 1 *Yin-Yang* Type Questionnaires**

| ***Yang* Type** | | | | | | | ***Yin* Type** | | | | | |
| --- | --- | --- | --- | --- | --- | --- | --- | --- | --- | --- | --- | --- |
|  | Most of the time | Good part of the time | Some of the time | A little of the time | None of the time | | | A little of the time | Some of the time | Good part of the time | Most of the time |  |
| Scores  *Yang*-  Subjective feelings | 8 | 6 | 4 | 2 | 0 | | | -2 | -4 | -6 | -8 | Scores  *Yin*-  Subjective feelings |
| I feel hot or hot flash, sweating at daily activities |  |  |  |  | 0 | | |  |  |  |  | I feel cold or chill occasionally, and always weakness with no sweating |
| I like to eat cool food and drink cool water |  |  |  |  | 0 | | |  |  |  |  | I like to eat hot food or drink hot water |
| I feel intolerance to hot environment |  |  |  |  | 0 | | |  |  |  |  | I feel intolerance to cold environment |
| I feel excessive thinking |  |  |  |  | 0 | | |  |  |  |  | I feel retardation of thinking |
| I feel quick in breathing |  |  |  |  | 0 | | |  |  |  |  | I feel weak in breathing |
| Scores  *Yang*-  Objective observation | 4 | 3 | 2 | 1 | 0 | | | -1 | -2 | -3 | -4 | Scores  *Yin*-  Objective observation |
| Redder complexion |  |  |  |  | 0 | | |  |  |  |  | Paler complexion |
| Redder tongue |  |  |  |  | 0 | | |  |  |  |  | Paler tongue |
| Dark urine |  |  |  |  | 0 | | |  |  |  |  | Clear urine |
| Constipation or dry stool |  |  |  |  | 0 | | |  |  |  |  | Loose or watery stool |
| Total |  | | | | |  | | | | | |  |

Note: definition of *Yang*: If *Yang* score > 32 and *Yin* score <8, means you could be *Yang* type; definition of *Yin*: If *Yin* score > 32 and *Yang* score <8, means you could be *Yin* type.
